# Motivational predictors and configurations associated with youths’ intention to share intangible cultural heritage knowledge on Douyin: a PLS-SEM and fsQCA study

**DOI:** 10.3389/fpsyg.2026.1935285

**Published:** 2026-10-20

**Authors:** Jia Hu, Shuhaida Md Noor, S. Mostafa Rasoolimanesh

**Affiliations:** 1 School of Journalism and Communication, Yangzhou University, Yangzhou, China; 2 School of Communication, Universiti Sains Malaysia, Pulau Pinang, Malaysia; 3 School of Business and Law, Edith Cowan University, Joondalup, WA, Australia

**Keywords:** configurational analysis, Douyin, intangible cultural heritage, knowledge sharing, youth motivation

## Abstract

Intangible cultural heritage (ICH) depends on the continued transmission of culturally embedded knowledge across generations, yet digital exposure does not necessarily translate into active knowledge sharing among young people. This study examines the motivational structure associated with young adults’ intention to share intangible cultural heritage knowledge (ICHK) on Douyin, a short-video platform in China. Drawing on the Motives, Values, and Preferences Inventory (MVPI), an exploratory qualitative pretest was first used to identify seven motivational domains that were contextually salient to Douyin-based ICHK sharing. Survey data from 400 eligible respondents aged 18–24 in Sichuan Province were subsequently analyzed using partial least squares structural equation modeling (PLS-SEM) and fuzzy-set qualitative comparative analysis (fsQCA). PLS-SEM was used to estimate the net associations between individual motives and sharing intention, whereas fsQCA was used to identify combinations of motivational conditions associated with high intention. The PLS-SEM results show that aesthetic, altruistic, commercial, affiliation, tradition, and recognition motives are positively associated with sharing intention, while hedonistic motive shows no significant independent net association. The fsQCA analysis identifies nine equifinal sufficient configurations in the baseline solution. Notably, hedonistic motive appears in seven configurations despite its nonsignificant PLS-SEM coefficient, illustrating the distinction between independent net relevance and configurational relevance. The findings suggest that digital heritage knowledge sharing is motivationally heterogeneous and context-dependent, with cultural, social, expressive, reputational, hedonic, and instrumental motives operating in different combinations. The study contributes to digital media psychology and cultural heritage communication by integrating a broad motivational taxonomy with complementary variable-centered and configurational perspectives.

## 1 Introduction

Intangible cultural heritage (ICH) depends on the continued transmission of culturally embedded knowledge, skills, practices, and meanings across generations (Zhang et al., 2026). More broadly, digital transformation research has demonstrated the growing role of digital technologies in enabling sustainable transformation across institutional contexts (Zhu et al., 2026). In the cultural domain, as traditional modes of transmission face pressures associated with social transformation and changing patterns of cultural participation, digital platforms have become increasingly important environments through which younger generations encounter and engage with heritage knowledge (Falk and Hagsten, 2025; Wahed et al., 2021). However, greater digital exposure does not necessarily translate into active participation. Young users may view, follow, or interact with heritage-related content without actively contributing to its circulation or explaining the knowledge embedded within it (Hu and Noor, 2026). This distinction is consequential because the sustainability of ICH depends not only on access to cultural content but also on continued knowledge transmission and participation by younger generations (Kim et al., 2019; Shimray and Ramaiah, 2022; Ren and Lam, 2026). Understanding what is associated with youths’ intention to actively share intangible cultural heritage knowledge (ICHK) is therefore important for explaining participation in contemporary digital heritage communication.

Existing research on social-media knowledge sharing has identified a wide range of motivational factors, including altruism, rewards, reputation, reciprocity, self-efficacy, enjoyment, and affiliation (Allam et al., 2020; Assegaff et al., 2016; Luo et al., 2020; Maheshwari et al., 2020; Pi et al., 2013; Safa and Von Solms, 2016; Todorova and Mills, 2018). Much of this evidence, however, has been developed in relation to platforms such as Facebook, WeChat, Zhihu, and Wiki, where knowledge exchange is commonly embedded in text-oriented communication or relatively stable social relationships (Cai et al., 2022; Jin et al., 2021; Lim et al., 2025; Pi et al., 2013; Ismail, 2020). Douyin provides a different communicative environment. Its short-video format foregrounds audiovisual expression, while recommendation-based distribution can extend content visibility beyond existing interpersonal networks and make audience responses observable through views, likes, comments, shares, and follower growth (Cao, 2025). These characteristics do not necessarily create new motives, but they may alter the opportunities through which existing motives are expressed, rewarded, and made behaviorally salient. Motivational relationships identified on conventional social-media platforms therefore cannot simply be assumed to operate in the same way when culturally embedded knowledge is shared through an algorithmically curated short-video environment (Anderson, 2020; Cao, 2025; Du et al., 2022).

A second unresolved issue concerns the type of knowledge being shared. ICHK differs from generic online information because it is embedded in cultural practices, collective memory, historical continuity, and intergenerational transmission (Buzeta et al., 2020). Its circulation may therefore involve familiar social-media motives such as enjoyment, recognition, affiliation, and economic reward while simultaneously invoking aesthetic appreciation, helping others understand cultural traditions, and a perceived commitment to cultural continuity. Previous studies have identified individual motivational factors associated with cultural-heritage knowledge sharing and digital engagement, including rewards, relational expectations, enjoyment of helping, reciprocity, edutainment, and environmental or experiential qualities (Shimray and Ramaiah, 2022; Di Leo, 2024). Nevertheless, these factors remain relatively fragmented across different theoretical perspectives. Consequently, existing research provides limited insight into how a broader set of motivational dispositions can be organized within a common framework when the object of sharing is culturally embedded heritage knowledge.

A third issue concerns how motivational relevance is conceptualized. Most studies of knowledge-sharing intention adopt a variable-centered perspective and examine whether individual predictors have statistically significant average relationships with behavioral intention (Nguyen et al., 2021). Such analyses are useful for estimating the independent association of a particular motive while accounting for other predictors, but they do not fully address whether different motives may complement or substitute for one another (Nguyen et al., 2019). This issue is particularly relevant to digital heritage participation, where a young user may share ICHK because of cultural responsibility and altruistic concern, whereas another may arrive at similarly high sharing intention through aesthetic expression, social recognition, affiliation, or legitimate instrumental incentives. Recent configurational research has shown that the effectiveness of ICH communication on Douyin can be associated with different combinations of platform characteristics, content attributes, and user-engagement conditions (Wang et al., 2025). However, the configurational structure of youths’ underlying motivations for actively sharing ICHK remains less clearly understood. It therefore remains necessary to distinguish between the independent net relevance of individual motives and their configurational relevance when they operate in conjunction with other motivations.

To address these issues, the present study adopts the Motives, Values, and Preferences Inventory (MVPI) as a broad motivational taxonomy and applies it contextually to youths’ ICHK-sharing intention on Douyin. Rather than assuming that all original MVPI dimensions are equally relevant across technological and cultural settings, an exploratory qualitative pretest was first used to assess which motivational domains were salient in this specific context. Seven dimensions—aesthetic, affiliation, altruistic, commercial, hedonistic, recognition, and tradition motives—were subsequently examined in the quantitative study. This context-sensitive use of the MVPI allows the study to investigate how broad motivational dispositions are expressed when culturally embedded knowledge is communicated through a visually oriented short-video platform, rather than merely transferring an established inventory to a new empirical setting.

The study then combines partial least squares structural equation modeling (PLS-SEM) and fuzzy-set qualitative comparative analysis (fsQCA) to examine two complementary aspects of motivational relevance. PLS-SEM is used to estimate the net associations between individual motivational predictors and youths’ intention to share ICHK, whereas fsQCA is used to identify combinations of motivational conditions associated with high sharing intention. The two approaches are therefore not treated as alternative tests expected to yield identical conclusions. Instead, their integration permits examination of whether a motive that is important on average is also recurrent within sufficient configurations and, conversely, whether a motive with limited independent net relevance may become salient when combined with other conditions. In this way, the study contributes to digital media psychology, social-media knowledge-sharing research, and cultural heritage communication by linking a broad motivational taxonomy with the cultural characteristics of ICHK, the communication environment of Douyin, and a configurational account of motivational heterogeneity.

## 2 Theoretical foundation

### 2.1 MVPI as a broad motivational taxonomy

Motivation stands as a pivotal determinant of human behavior, influencing an individual’s actions, choices, and achievements (Nguyen et al., 2021). Scholars have formulated various theories to elucidate motivation, including the hierarchy of needs, achievement motivation, two-factor theory, goal-set theory, expectancy theory, attribution theory, and self-determination theory, aiming to understand the drivers of individuals’ pursuits and successes across diverse domains. Each theory offers a unique perspective on human motivation, unveiling intricate psychological mechanisms that influence individual behavior. Drawing from these theoretical frameworks, several assessment tools have been devised to discern key motivational dimensions along the intrinsic-extrinsic spectrum. The MVPI stands out for directly assessing individual motives (Furnham, 2021). Developed by Hogan and Hogan (2010), the MVPI integrates insights from eight decades of motivation, values, and interest research, encompassing 10 motives/preferences, including aesthetic motive, altruistic motive, commercial motive, hedonistic motive, power motive, recognition motive, scientific motive, security motive, affiliation motive, and tradition motive. Supplementary Table A1 illustrates the definition of each motivation.

To achieve a comprehensive understanding of the theoretical underpinnings, empirical substantiation, and pragmatic application of MVPI across various domains, a thorough review of the extant literature was conducted. As shown in Supplementary Table A2, most scholars directly use the original measurement dimensions of MVPI in their research, and the focus is mainly on the aspects of psychometric characteristics and validation (Furnham, 2021; Furnham and Treglown, 2021; Romanová et al., 2016), individual differences and personality (Furnham et al., 2015; Furnham and Treglown, 2022; Jordan et al., 2018), and organizational behavior and leadership (Bălan et al., 2019; Furnham et al., 2016; Noaghea et al., 2017). The framework therefore offers a sufficiently broad motivational vocabulary for examining heterogeneous reasons for action, but it was not developed specifically for social-media knowledge sharing or cultural heritage communication.

This distinction is theoretically important. Applying the MVPI to ICHK sharing does not imply that all 10 original dimensions should necessarily exhibit equal behavioral relevance in every technological or knowledge context. The original taxonomy defines the available motivational domains; the empirical salience of those domains may vary according to the behavior under investigation. In the present study, the MVPI is therefore treated as a broad theoretical taxonomy rather than as a fixed 10-factor behavioral model that must be reproduced unchanged in every setting.

### 2.2 Contextualizing motivation in Douyin-based ICHK sharing

Knowledge-sharing research provides empirical support for several motivational domains that conceptually correspond to the broader MVPI taxonomy. Previous studies have identified altruistic helping, rewards, reputation, enjoyment, and affiliation as relevant to knowledge-sharing intention across online environments (Allam et al., 2020; Bakshi et al., 2019; Luo et al., 2020; Maheshwari et al., 2020; Todorova and Mills, 2018; Zhao and Detlor, 2023). Other studies have also examined inhibiting considerations related to security or the perceived loss of knowledge power (Moghavvemi et al., 2017; Oliveira et al., 2020). These findings suggest that knowledge sharing is motivationally heterogeneous rather than reducible to a single intrinsic or extrinsic incentive.

ICHK sharing introduces an additional layer of contextual specificity because the knowledge being communicated is culturally embedded. ICH consists not only of information but also of practices, meanings, skills, identities, and forms of collective memory that depend on continued social transmission (Zhang et al., 2026). Sharing such knowledge can therefore involve motivations associated with cultural appreciation, helping others understand heritage, moral commitment to cultural continuity, social connection, recognition as a cultural communicator, enjoyment of creative expression, and potential material rewards. Consequently, the behavioral relevance of a motivational domain depends not only on the general act of knowledge sharing but also on the cultural meaning attached to the knowledge being shared.

Platform context provides a second source of contextual differentiation. Research on digital heritage communication has shown that social media, livestreaming, short video, and algorithmically curated platforms structure heritage visibility, audience interaction, and content circulation in different ways (Cao, 2025; Lu et al., 2019; Hammou et al., 2020; Paquienseguy and Guo, 2025; Wang et al., 2025). On Douyin, audiovisual production creates opportunities for aesthetic expression; visible audience feedback provides opportunities for recognition; interaction around shared interests enables affiliation; and the creator economy provides potential instrumental or commercial incentives. These affordances should not be interpreted as generating the underlying motives themselves. Rather, they provide different conditions through which existing motives may be enacted and become behaviorally salient.

The theoretical relationship can therefore be expressed as a process of contextual motivational salience: broad motivational dispositions provide potential reasons for action, while the characteristics of the knowledge domain and communication environment shape which of these reasons become particularly relevant to a specific behavior. In the present context, the behavior is not generic social-media participation but the intentional sharing of culturally embedded knowledge through a visually oriented short-video platform.

### 2.3 Conceptual differentiation of the retained motives

Because several motives may coexist within the same sharing decision, conceptual differentiation is necessary before examining their empirical relationships. In this study, aesthetic motive refers to the value attached to self-expression, cultural form, and aesthetic experience; its defining concern is the expressive and sensory quality of the activity. Altruistic motive is other-oriented and concerns the perceived value of helping others acquire or understand knowledge. Affiliation motive concerns social connection, interaction, and belonging, whereas recognition motive concerns attention, approval, prestige, and favorable evaluation by others. The distinction between these two social motives is therefore relational connection versus evaluative status. Tradition motive is conceptually different from altruism and affiliation because it concerns adherence to moral, normative, and culturally established values rather than helping particular others or forming social relationships. In ICHK sharing, it is associated with a perceived obligation to contribute to the continuity of cultural knowledge. Commercial motive, by contrast, reflects instrumental interest in economic reward and value creation. Finally, hedonistic motive concerns enjoyment, pleasure, excitement, and fun arising from the sharing activity itself. These dimensions may correlate empirically, but they represent theoretically distinct valued outcomes and therefore need not produce identical relationships with sharing intention.

Before the formal survey, an exploratory qualitative pretest was conducted to assess which MVPI domains were contextually salient in accounts of Douyin-based ICHK sharing. The purpose of this procedure was not to redefine the original MVPI taxonomy but to determine which domains were sufficiently relevant to the focal behavior to warrant inclusion in the empirical model. Seven domains—aesthetic, affiliation, altruistic, commercial, hedonistic, recognition, and tradition motives—were retained. Scientific, power, and security motives were not spontaneously identified in participants’ open-ended accounts and were therefore treated as contextually non-salient for the present study rather than as universally irrelevant motivations. The participant characteristics, interview procedure, coding process, and retention criteria are reported in the Methodology section.

## 3 Hypotheses development

### 3.1 Aesthetic motive

The aesthetic motive reflects a preference for artistic expression, cultural form, and visually appealing experiences (Hogan and Hogan, 2010). Aesthetic considerations are particularly relevant to ICHK because many heritage practices are communicated through visible forms such as performance, craftsmanship, dress, ritual, movement, and material detail (Liu and Khairul, 2025). In digital storytelling, visual presentation can support cultural legibility and audience engagement, while aesthetic value has been associated with participation in cultural content on social media (Liu and Khairul, 2025; Villaespesa and Wowkowych, 2020; Xie et al., 2024). Douyin provides a communication environment in which such preferences can be enacted through short-form audiovisual production, including composition, editing, music, movement, and visual storytelling (Saidkulovich and Ugli, 2021). Its recommendation-based distribution can also expose visually presented heritage content beyond users’ immediate social networks (Du et al., 2022). These characteristics do not imply that the platform creates aesthetic motivation; rather, they provide an environment in which aesthetic expression may become particularly relevant to ICHK sharing. Young users who place greater value on aesthetic expression may therefore be more inclined to regard sharing ICHK on Douyin as a meaningful form of cultural and self-expression. Hence, the hypothesis posits:

*H1*. Aesthetic motive is positively associated with youths’ intention to share ICHK on Douyin.

### 3.2 Altruistic motive

The altruistic motive refers to a preference for helping others and contributing to their welfare without requiring direct personal reward (Hogan and Hogan, 2010; Munar and Jacobsen, 2014). Altruism has been consistently associated with knowledge-sharing activities in online communities and social-media environments, where individuals may contribute information because they believe it will benefit other users (Fang and Zhang, 2019; Allam et al., 2020; Bakshi et al., 2019; Yuan and Liu, 2017; Zhao and Detlor, 2023). This motivation has particular relevance to ICHK sharing because heritage knowledge may be understood as a cultural resource whose continuity depends partly on being explained, documented, and made accessible to others. Sharing information about a heritage practice can help audiences understand its techniques, meanings, histories, or cultural context and can contribute to broader public awareness. Douyin enables such knowledge to reach audiences beyond immediate interpersonal networks through accessible short-form content. For young users who value helping others understand cultural heritage, sharing ICHK may therefore constitute a form of prosocial knowledge contribution. Therefore, the following hypothesis is posited:

*H2*. Altruistic motive is positively associated with youths’ intention to share ICHK on Douyin.

### 3.3 Hedonistic motive

The hedonistic motive reflects the value individuals place on enjoyment, pleasure, excitement, and fun (Hogan and Hogan, 2010). Enjoyment has been associated with technology use and online knowledge-sharing activities, particularly where participation itself provides an intrinsically rewarding experience (Al-Azawei and Alowayr, 2020; Al-Emran et al., 2023; Cai et al., 2022; Dong et al., 2024; Kahil, 2021; Liu and Luo, 2023). Sharing ICHK through short video may involve creative activities such as selecting cultural material, developing a narrative, editing audiovisual content, combining music and imagery, and interacting with audiences. These processes can make cultural knowledge sharing an experiential and creative activity rather than a purely informational task (Paquienseguy and Guo, 2025). Douyin’s short-form audiovisual environment therefore provides opportunities through which enjoyment and creative engagement may become relevant to the sharing process. Young users who derive greater pleasure from producing and communicating cultural content may consequently report stronger intentions to participate in ICHK sharing. Consequently, the subsequent hypothesis is proposed:

*H3*. Hedonistic motive is positively associated with youths’ intention to share ICHK on Douyin.

### 3.4 Commercial motive

The commercial motive reflects an orientation toward financial return, economic value, business opportunity, and material reward (Hogan and Hogan, 2010). Prior knowledge-sharing research has shown that tangible rewards and economic incentives can be associated with individuals’ willingness to contribute knowledge in online environments (Maheshwari et al., 2020; Shimray and Ramaiah, 2022; Todorova and Mills, 2018; Wang et al., 2022). The relevance of commercial motivation to ICHK sharing arises from the increasing integration of cultural content into digital creator economies. Short-video production can involve substantial time and creative effort, while successful cultural content may generate opportunities related to traffic incentives, cultural-product promotion, creator collaborations, or other forms of economic return (China Daily, 2019). In this context, economic motivation need not be interpreted as incompatible with cultural communication. Rather, material return can coexist with cultural, social, or expressive reasons for participation. Young users who place greater value on economic and instrumental outcomes may therefore show stronger intentions to contribute ICHK through Douyin. Hence, the following hypothesis is posited:

*H4*. Commercial motive is positively associated with youths’ intention to share ICHK on Douyin.

### 3.5 Affiliation motive

The affiliation motive reflects a preference for interpersonal connection, frequent social interaction, and a sense of belonging (Hogan and Hogan, 2010; Zhou et al., 2020). Knowledge sharing can provide opportunities to establish or strengthen relationships with individuals who have common interests, and previous research has associated affiliation motivation with knowledge-sharing participation on social media (Luo et al., 2020; Zhao and Detlor, 2023). ICHK provides a potential basis for interest-oriented social interaction because users who share concern for particular traditions, crafts, performances, communities, or cultural practices may interact around common cultural interests. On Douyin, comments, following, sharing, and creator–audience interaction can facilitate connections among users who may not have pre-existing offline relationships. Accordingly, the relevance of affiliation differs from that of recognition: affiliation concerns the formation and maintenance of social connection, whereas recognition concerns favorable evaluation and status. Young users who value social connection may therefore be more inclined to share ICHK when doing so provides opportunities to interact with culturally interested others. Therefore, the subsequent hypothesis is proposed:

*H5*. Affiliation motive is positively associated with youths’ intention to share ICHK on Douyin.

### 3.6 Tradition motive

The tradition motive reflects a preference for established values, normative standards, moral commitment, and continuity with culturally embedded ways of life (Hogan and Hogan, 2010). In knowledge-sharing contexts, moral obligation can provide a reason for contributing information when individuals regard knowledge as a collective or public good (Xu et al., 2020; Steinberg, 2020). This motive is especially relevant to ICHK because intangible heritage consists of living practices and knowledge whose continuity depends on repeated transmission, interpretation, and participation across generations (Dippon and Moskaliuk, 2020; Kim et al., 2019). Sharing ICHK may therefore be understood by some young users as more than an act of online content production; it can also represent a culturally or morally meaningful contribution to maintaining knowledge about inherited practices. This distinguishes tradition motive from altruism: altruism centers on benefiting others, whereas tradition centers on consistency with normative and cultural commitments. Consequently, this hypothesis is proposed:

*H6*. Tradition motive is positively associated with youths’ intention to share ICHK on Douyin.

### 3.7 Recognition motive

The recognition motive reflects a preference for attention, approval, acknowledgement, prestige, and favorable evaluation by others (Hogan and Hogan, 2010; Benedek et al., 2020). Recognition has been associated with online knowledge sharing because contributing visible or valued information can strengthen users’ perceived reputation and social standing (Liu and Luo, 2023; Luo et al., 2020; Todorova and Mills, 2018). Recognition may acquire particular relevance on short-video platforms because audience evaluation is highly visible. Views, likes, comments, follower growth, featured content, and other observable responses can provide users with signals of how their contributions are received. In the case of ICHK, sharing culturally informative content may also allow users to be perceived as knowledgeable or engaged cultural communicators (Wang et al., 2025). These platform conditions do not establish that recognition necessarily causes sharing; rather, they provide observable opportunities through which recognition-oriented users may experience social acknowledgement. Young users who place greater value on such acknowledgement may therefore report stronger intentions to share ICHK. Hence, the following hypothesis is formulated:

*H7*. Recognition motive is positively associated with youths’ intention to share ICHK on Douyin.

## 4 Conceptual framework

This study employs a dual-methodological approach, integrating SEM and fsQCA, to comprehensively examine the factors influencing youths’ intention to share ICHK via Douyin.

SEM is a widely recognized statistical technique used to analyze linear causal relationships between variables and to quantitatively test theoretical frameworks (Hair et al., 2019). In this study, SEM is utilized to validate the direct effects of various motivational factors on the intention to share ICHK via Douyin. These factors include aesthetic motive, affiliation motive, altruistic motive, commercial motive, hedonistic motive, recognition motive, and tradition motive. SEM provides a robust framework for confirming hypothesised relationships and quantitatively assessing their strength. However, SEM assumes data homogeneity and may not fully capture potential nonlinear or configurational interactions among variables (Hair et al., 2019).

To complement the linear analysis provided by SEM, this study also applies fsQCA, a method grounded in complexity theory. Complexity theory posits that social phenomena are shaped by multiple interdependent factors that interact in nonlinear, asymmetric ways (Byrne, 2005). Unlike traditional regression-based methods, fsQCA allows the identification of numerous causal pathways and accounts for configurational interactions among variables (Ragin, 2008). The equifinality principle of complexity theory further supports the idea that different configurations of motives can lead to the same outcome (Woodside, 2014). Thus, by integrating SEM and fsQCA, this study provides a more holistic understanding of the motivational factors underlying ICHK sharing (see Figure 1). The SEM model provides a quantitative assessment of the direct effects of the identified motivational factors. The fsQCA model illustrates the configurational relationships and multiple causal pathways that influence the intention to share ICHK via Douyin. This dual-methodological approach allows for a comprehensive analysis that combines the strengths of both quantitative and configurational methods, providing a richer and more nuanced understanding of the complex motivational dynamics at play in ICHK sharing among youths.

**Figure 1:**
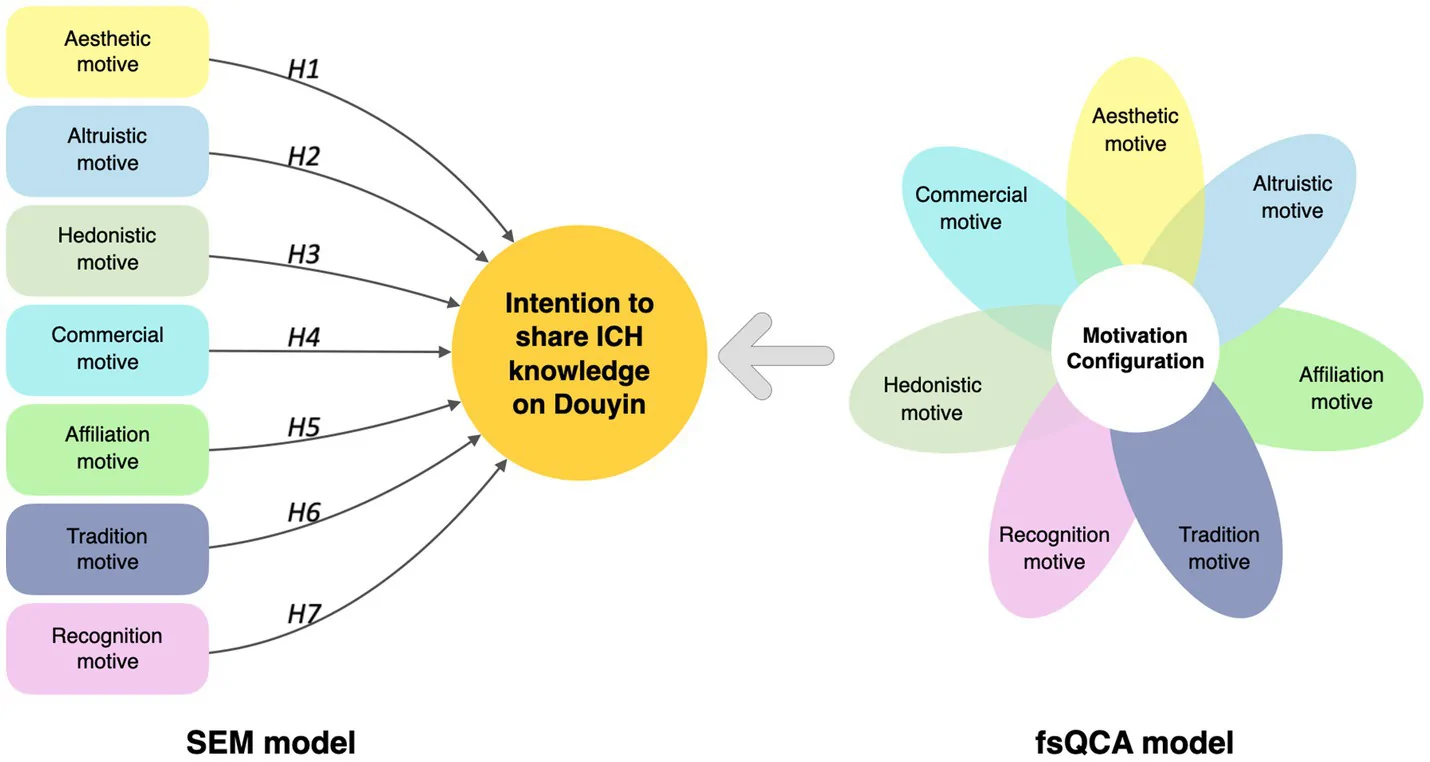
Conceptual model.

## 5 Methodology

### 5.1 Qualitative pretest

Before the formal survey, an exploratory qualitative pretest was conducted to assess the contextual salience of the 10 motivational domains represented in the MVPI for Douyin-based ICHK sharing. The purpose of this pretest was not to redefine the original MVPI taxonomy but to determine which motivational domains were sufficiently relevant to the focal behavior to warrant inclusion in the subsequent quantitative model.

24 young participants aged 18–24 were recruited from the four study regions: Chengdu, Aba, Ganzi, and Liangshan. Six participants were recruited from each region, with three males and three females per region, resulting in an overall gender distribution of 12 males and 12 females. All participants met the eligibility criteria subsequently applied in the formal survey: they were aged 18–24, resided in one of the four study regions, had active experience using Douyin, and possessed basic awareness of ICH.

Individual face-to-face interviews were conducted, each lasting approximately 30–45 min. With participants’ consent, the interviews were audio-recorded and transcribed verbatim. The interview protocol followed a two-stage sequence. Participants were first asked open-ended questions about their experiences of Douyin, their understanding of ICH, and their reasons for being willing or unwilling to share ICHK. These open-ended questions were intentionally presented before any theory-informed prompts so that participants could articulate motivational considerations in their own terms. The second stage used probes corresponding to the MVPI domains to explore the relevance of motivational themes that might not have emerged spontaneously. The interview guide is provided in the Supplementary Material.

Two researchers independently reviewed and coded the interview transcripts. Initial coding focused on statements indicating reasons, expected benefits, concerns, values, or anticipated outcomes associated with ICHK sharing. These codes were then compared with the 10 MVPI domains. Coding disagreements were discussed until agreement was reached. No numerical inclusion threshold was predetermined before the interviews; instead, the decision focused primarily on whether a motivational domain emerged spontaneously and recurrently in participants’ accounts. Each of the seven retained domains was spontaneously mentioned by at least two participants.

The pretest indicated recurrent contextual relevance for aesthetic, affiliation, altruistic, commercial, hedonistic, recognition, and tradition motives. By contrast, scientific, power, and security motives were not spontaneously identified in participants’ open-ended accounts. These three motives were therefore treated as contextually non-salient for the present focal behavior and were not included in the quantitative model. Their exclusion should not be interpreted as evidence that these motives are universally irrelevant to cultural heritage sharing; rather, the pretest indicated limited salience in this specific population, platform, and behavioral context.

### 5.2 Participants, sampling, and data collection

The formal survey was administered online through Wenjuanxing between July 30 and October 30, 2023. Before accessing the questionnaire, respondents were informed of the research purpose, voluntary nature of participation, anonymity, confidentiality, and right to withdraw. Electronic informed consent was required before respondents could proceed. The study protocol was approved by the Human Research Ethics Committee of Universiti Sains Malaysia (Approval No. USM/JEPeM/PP/23040337) and was conducted in accordance with the Declaration of Helsinki.

Participants were required to satisfy four eligibility criteria. First, they had to be aged 18–24 years. Second, they had to reside in Chengdu, Aba, Ganzi, or Liangshan in Sichuan Province. Third, they had to qualify as active Douyin users. Active use was operationalized as participation in at least one of three forms of platform activity: contribution activities, such as liking, commenting, forwarding, following, or saving content; enhancement activities, such as editing videos, applying filters or transitions, adding music or subtitles; or creation activities, such as producing videos, livestreaming, or interacting with followers. Respondents who were not Douyin users or who reported only viewing videos were excluded. Fourth, respondents were required to demonstrate basic ICH awareness.

Basic ICH awareness was operationalized using a screening item assessing familiarity with ICH elements relevant to the study context. Respondents rated their familiarity on a five-point scale ranging from 1 (poor) to 5 (very good). A respondent was eligible to continue if a score of at least 3 was reported for at least one listed ICH element; respondents who did not meet this criterion were screened out. The formal questionnaire presented respondents with the official Chinese definition of ICH before this screening procedure.

Because no complete sampling frame was available and eligible participants were geographically dispersed, exponential non-discriminative snowball sampling was employed. Initial participants were recruited through multiple pre-existing WeChat networks, including university associations, volunteer networks, and cultural-interest groups, across the four regions. These participants subsequently referred other eligible respondents, allowing recruitment to expand across multiple referral networks rather than a single institutional source.

Equal regional quotas were used to ensure that each of the four study areas contributed a sufficient and analytically comparable number of observations and to reduce the possibility that recruitment would be dominated by a single, more accessible regional network. The target was 100 valid responses per region. These quotas were not population-proportional and were not intended to make the sample statistically representative of the youth populations of the four regions.

A total of 500 questionnaires were collected. After application of the eligibility and data-quality criteria, 400 valid cases were retained for analysis, corresponding to 100 respondents from each region. Cases were excluded if respondents fell outside the target age range, did not meet the active-Douyin-use criterion, did not satisfy the ICH-awareness screening criterion, resided outside the four study regions, or provided incomplete or inconsistent responses.

#### 5.2.1 Sample characteristics

The final sample consisted of 400 participants (see Table 1). Males accounted for 52.8% of the sample (*n* = 211) and females for 47.3% (*n* = 189). Most respondents were aged 18–21 years (83.8%, *n* = 335), whereas 16.3% (*n* = 65) were aged 22–24 years. Regarding education, 74.0% (*n* = 296) were in the college/university category, 15.5% (*n* = 62) were in secondary/high school, 9.8% (*n* = 39) were postgraduates, and 0.8% (*n* = 3) reported another educational status. By design, Chengdu, Aba, Ganzi, and Liangshan each contributed 100 respondents (25.0%). In terms of Douyin-use experience, 74 respondents (18.5%) were categorized as participating in contribution activities, 149 (37.3%) in enhancement activities, and 177 (44.3%) in creation activities.

**Table 1:** Characteristics of the final sample (*n* = 400).

Characteristic	Category	*n*	%
Gender	Male	211	52.8
	Female	189	47.3
Age	18–21	335	83.8
	22–24	65	16.3
Education	Secondary/high school	62	15.5
	College/university	296	74.0
	Postgraduate	39	9.8
	Other/none	3	0.8
Region	Chengdu	100	25.0
	Aba	100	25.0
	Ganzi	100	25.0
	Liangshan	100	25.0
Douyin-use experience	Contribution activities	74	18.5
	Enhancement activities	149	37.3
	Creation activities	177	44.3

### 5.3 Measurement development and content validation

Measurement items for the eight constructs were adapted from previously validated scales (see Supplementary Table A3). Aesthetic motive was measured using three items adapted from Snelgrove et al. (2008), affiliation motive using four items from Jeon et al. (2011), altruistic motive using three items from Hsu and Lin (2008), commercial motive using three items from Wei et al. (2010), hedonistic motive using three items from Hsu and Lin (2008), recognition motive using five items from Kankanhalli et al. (2005), tradition motive using three items from Lai and Hsieh (2013), and intention to share ICHK on Douyin using three items from Hung et al. (2015).

The items were contextually adapted so that the referent behavior consistently concerned sharing ICHK on Douyin. Adaptation focused on construct–item fit, relevance to Douyin-based ICHK sharing, clarity of wording, ambiguity, and equivalence between the English and Chinese versions. The underlying conceptual meaning of each construct was retained while the behavioral referent was made specific to the study context.

Content validation was conducted by four experts with academic expertise in communication, knowledge management, ICH/cultural heritage, psychology, social media, measurement, or bilingual questionnaire adaptation. The panel included professors and associate professors with empirical research experience related to youth behavior. Experts evaluated the correspondence between each item and its intended construct, relevance to Douyin-based ICHK sharing, contextual appropriateness, wording clarity, potential ambiguity, and bilingual consistency. Written item-level comments were collected and further discussed through face-to-face or online consultation where clarification was required.

Item relevance was evaluated on a four-point scale and summarized using the item-level content validity index (I-CVI) and the scale-level content validity index based on the average method (S-CVI/Ave). I-CVI values ranged from 0.75 to 1.00, and the S-CVI/Ave was 0.99. Items receiving relatively lower agreement were reconsidered through expert discussion and were revised or removed where necessary rather than being retained automatically.

Expert review resulted in several contextual and linguistic revisions. For example, the midpoint response label was revised from “neutral” to “neither agree nor disagree” to reduce ambiguity; the questionnaire introduction adopted the official Chinese definition of ICH for the formal Sichuan survey; and the age question was converted from an open response format into predefined screening categories. The Douyin-use item was also refined to distinguish passive viewing from contribution, enhancement, and creation activities. Items judged redundant or insufficiently aligned with the focal construct were reconsidered and removed where appropriate.

The instrument was initially developed in English and translated into Chinese. Two bilingual experts reviewed and refined the Chinese wording, after which two independent bilingual experts conducted back-translation into English. The original and back-translated versions were compared for semantic consistency, and discrepancies were discussed until agreement was reached. The final questionnaire was administered in Chinese.

Because predictor and outcome variables were collected from the same respondents using the same questionnaire, common method bias was assessed using two *post hoc* diagnostics. Harman’s single-factor test indicated that the first unrotated factor accounted for 32.68% of the total variance. In addition, full-collinearity VIF values ranged from 1.417 to 2.879, below the 3.3 criterion. Taken together, these diagnostics did not indicate substantial common method bias, although *post hoc* statistical tests cannot completely rule out common method variance.

### 5.4 Data analysis

#### 5.4.1 PLS-SEM

PLS-SEM was conducted using WarpPLS 8.0. The method was selected because the study has an explanatory–predictive orientation and seeks to estimate the relative net associations of multiple motivational predictors with sharing intention, while also providing a variable-centered counterpart to the configurational analysis. The choice was therefore based primarily on the analytical objective rather than on sample-size limitations.

A two-stage assessment procedure was followed (Hair et al., 2019). First, the measurement model was evaluated in terms of indicator reliability, internal consistency reliability, convergent validity, and discriminant validity. Internal consistency was assessed using Cronbach’s alpha and composite reliability, convergent validity using average variance extracted (AVE), and discriminant validity using the heterotrait–monotrait ratio (HTMT), with values below 0.90 used as the criterion.

Second, the structural model was evaluated using collinearity diagnostics, the coefficient of determination (*R*^2^ and adjusted *R*^2^), path-specific effect sizes, predictive relevance (*Q*^2^), and the magnitude and statistical significance of the structural coefficients. Statistical inference was based on 5,000 bootstrap resamples, with bias-corrected and accelerated 95% confidence intervals. Out-of-sample predictive performance was additionally assessed using the PLSpredict procedure by comparing PLS-SEM prediction errors with those obtained from a linear-model benchmark.

#### 5.4.2 fsQCA

The seven motivational conditions and the outcome were calibrated into fuzzy sets using the direct calibration method. Because no externally established substantive thresholds were available for these multi-item motivational constructs, sample-relative anchors were adopted. The 95th percentile was used to indicate full membership, the 50th percentile as the crossover point, and the 5th percentile as full non-membership (Ragin, 2008). The 95th and 5th percentiles were used rather than the sample maximum and minimum to reduce sensitivity to extreme observations, while the median provided a transparent sample-relative point of maximum membership ambiguity.

For the sufficiency analysis, the truth table was constructed using a frequency threshold of three cases, a raw-consistency threshold of 0.80, and a PRI-consistency threshold of 0.80. Truth-table rows meeting these criteria were retained for logical minimization.

## 6 Results

### 6.1 PLS-SEM result

#### 6.1.1 Measurement model

The measurement model was evaluated in terms of indicator reliability, internal consistency reliability, convergent validity, and discriminant validity. As shown in Table 2, all indicator loadings exceeded 0.70 (Hair et al., 2019), ranging from 0.719 to 0.911. Cronbach’s alpha values ranged from 0.700 to 0.839, and composite reliability values ranged from 0.834 to 0.886, indicating satisfactory internal consistency across the eight constructs. Average variance extracted (AVE) values ranged from 0.581 to 0.702 and therefore exceeded the recommended 0.50 criterion, supporting convergent validity.

**Table 2:** Measurement model assessment.

Construct/item	Loading	Cronbach’s *α*	CR	AVE
Aesthetic motive		0.741	0.853	0.659
AESM1	0.803			
AESM2	0.841			
AESM3	0.791			
Affiliation motive		0.758	0.847	0.581
AFFM1	0.732			
AFFM2	0.836			
AFFM3	0.719			
AFFM4	0.759			
Altruistic motive		0.724	0.845	0.646
ALTM1	0.791			
ALTM2	0.853			
ALTM3	0.764			
Commercial motive		0.724	0.845	0.645
COMM1	0.804			
COMM2	0.840			
COMM3	0.763			
Hedonistic motive		0.700	0.834	0.626
HEDM1	0.806			
HEDM2	0.831			
HEDM3	0.734			
Recognition motive		0.839	0.886	0.610
RECM1	0.799			
RECM2	0.775			
RECM3	0.720			
RECM4	0.798			
RECM5	0.809			
Tradition motive		0.765	0.865	0.681
TRAM1	0.830			
TRAM2	0.827			
TRAM3	0.818			
Intention to share ICHK on Douyin		0.786	0.876	0.702
INT1	0.806			
INT2	0.911			
INT3	0.793			

CR,composite reliability; AVE,average variance extraction.

Discriminant validity was assessed using the heterotrait–monotrait ratio (HTMT; see Table 3). All HTMT values were below the 0.90 criterion (Henseler et al., 2015). The highest value was 0.866 between altruistic motive and intention to share ICHK, indicating that the constructs remained empirically distinguishable despite their theoretical relatedness. The measurement model therefore demonstrated satisfactory reliability, convergent validity, and discriminant validity.

**Table 3:** Heterotrait–monotrait ratios (HTMT) for discriminant validity.

Construct	AESM	AFFM	ALTM	COMM	HEDM	RECM	TRAM	INT
AESM								
AFFM	0.531							
ALTM	0.698	0.530						
COMM	0.623	0.539	0.627					
HEDM	0.747	0.378	0.578	0.525				
RECM	0.676	0.518	0.666	0.638	0.576			
TRAM	0.632	0.517	0.643	0.560	0.464	0.616		
INT	0.831	0.652	0.866	0.762	0.601	0.773	0.729	

All values are below the 0.90 criterion. AESM, aesthetic motive; AFFM, affiliation motive; ALTM, altruistic motive; COMM, commercial motive; HEDM, hedonistic motive; RECM, recognition motive; TRAM, tradition motive; INT, intention to share ICHK on Douyin.

#### 6.1.2 Structural model

Prior to interpreting the structural coefficients, collinearity was assessed. As shown in Figure 2, the full-collinearity VIF values ranged from 1.417 to 2.879, remaining below the conservative threshold of 3.3 and indicating no substantial collinearity concerns. The seven motivational predictors jointly explained 65.3% of the variance in intention to share ICHK on Douyin (R^2^ = 0.653; adjusted R^2^ = 0.646). Stone–Geisser’s Q^2^ value for sharing intention was 0.680, indicating predictive relevance.

**Figure 2:**
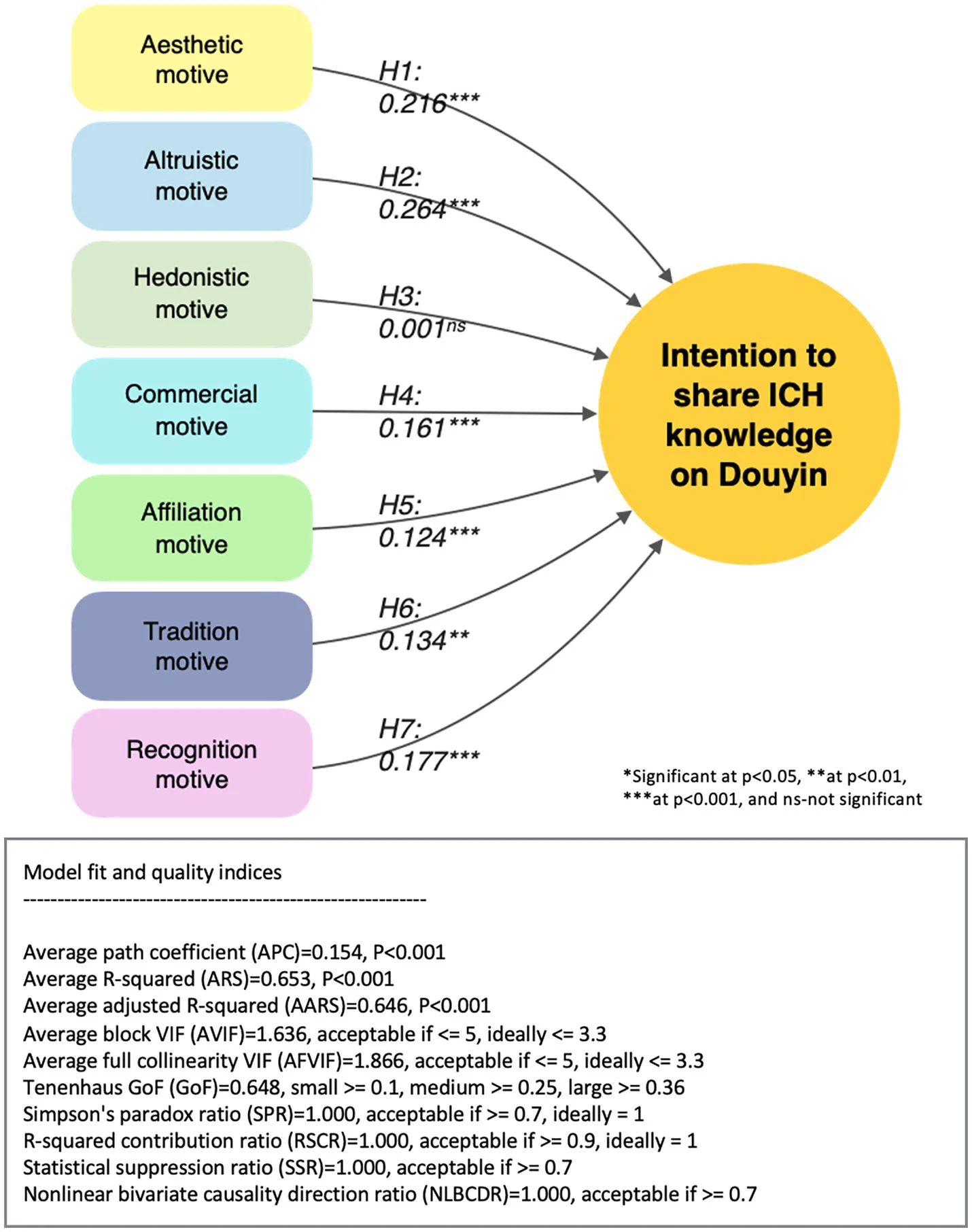
Results for a structural model.

Path-specific effect sizes were 0.155 for aesthetic motive, 0.057 for affiliation motive, 0.173 for altruistic motive, 0.099 for commercial motive, 0.001 for hedonistic motive, 0.124 for recognition motive, and 0.069 for tradition motive. Using conventional benchmarks (Kock, 2025), aesthetic and altruistic motives showed medium effect sizes; affiliation, commercial, recognition, and tradition motives showed small effect sizes; and the effect size of hedonistic motive was negligible.

Out-of-sample predictive performance was further assessed using the PLSpredict procedure (Hair et al., 2019). All three indicators of sharing intention yielded positive *Q*^2^ predict values (0.318–0.524; see Table 4). In addition, the PLS-SEM prediction errors were lower than those of the linear-model benchmark for all three indicators in terms of both RMSE and MAE. Specifically, PLS-SEM RMSE values ranged from 0.441 to 0.666, compared with 0.451 to 0.684 for the linear model. These results indicate high out-of-sample predictive performance according to the PLSpredict benchmark.

**Table 4:** PLSpredict assessment of out-of-sample predictive performance.

Indicator	*Q*^2^ predict	PLS-SEM RMSE	PLS-SEM MAE	LM RMSE	LM MAE
INT1	0.503	0.468	0.339	0.486	0.353
INT2	0.524	0.441	0.336	0.451	0.350
INT3	0.318	0.666	0.453	0.684	0.470

Positive *Q*^2^predict values indicate predictive relevance for the holdout observations. Lower PLS-SEM RMSE and MAE values relative to the linear-model (LM) benchmark indicate superior out-of-sample predictive performance. RMSE = root mean squared error; MAE = mean absolute error; LM = linear model.

Statistical inference was based on 5,000 bootstrap resamples with bias-corrected and accelerated 95% confidence intervals (see Table 5). Aesthetic motive was positively associated with sharing intention (*β* = 0.216, *p* < 0.001), as were altruistic motive (*β* = 0.264, *p* < 0.001), commercial motive (*β* = 0.161, *p* < 0.001), affiliation motive (*β* = 0.124, *p* < 0.001), tradition motive (*β* = 0.134, *p* = 0.003), and recognition motive (*β* = 0.177, *p* < 0.001). Hedonistic motive showed no significant net association with sharing intention (*β* = 0.001, *p* = 0.484). Accordingly, H1, H2, H4, H5, H6, and H7 were supported, whereas H3 was not supported.

**Table 5:** Structural model results.

H	Relationship	Beta (*β*)	*p*-value	95% Bca CI		Effect size	Decision
				LB	UB		
H1	AESM → INT	0.216	<0.001	0.117	0.316	0.155	Supported
H2	ALTM → INT	0.264	<0.001	0.180	0.347	0.173	Supported
H3	HEDM → INT	0.001	0.484	−0.065	0.067	0.001	Not Supported
H4	COMM → INT	0.161	<0.001	0.085	0.238	0.099	Supported
H5	AFFM → INT	0.124	<0.001	0.057	0.191	0.057	Supported
H6	TRAM → INT	0.134	0.003	0.039	0.229	0.069	Supported
H7	RECM → INT	0.177	<0.001	0.077	0.278	0.124	Supported

Bootstrap resamples = 5,000. AESM, aesthetic motive; AFFM, affiliation motive; ALTM, altruistic motive; COMM, commercial motive; HEDM, hedonistic motive; RECM, recognition motive; TRAM, tradition motive; INT, intention to share ICHK on Douyin.

### 6.2 fsQCA results

#### 6.2.1 Necessary condition analysis

Before the analysis, the seven motivational conditions and the outcome were calibrated into fuzzy-set membership scores. In the baseline specification, the 95th percentile was used as the threshold for full membership, the 50th percentile as the crossover point, and the 5th percentile as the threshold for full non-membership (Ragin, 2008). The actual calibration anchors for each condition and the outcome are reported in Table 6. The full-membership anchors ranged from 4.67 to 5.00, the crossover anchors from 3.67 to 4.00, and the full non-membership anchors from 3.00 to 3.33.

**Table 6:** Calibration anchors for the fsQCA analysis.

Calibration anchor	AESM	AFFM	ALTM	COMM	HEDM	RECM	TRAM	INT
Full membership (95th percentile)	5.00	5.00	5.00	5.00	4.67	5.00	5.00	5.00
Crossover (50th percentile)	4.00	4.00	4.00	4.00	3.67	4.00	4.00	4.00
Full non-membership (5th percentile)	3.00	3.00	3.33	3.00	3.00	3.00	3.00	3.00

AESM, aesthetic motive; AFFM, affiliation motive; ALTM, altruistic motive; COMM, commercial motive; HEDM, hedonistic motive; RECM, recognition motive; TRAM, tradition motive; INT, intention to share ICHK on Douyin.

Necessity analysis was then conducted to assess whether any individual condition or its negation was necessary for high sharing intention. A consistency threshold of 0.90 was used as the criterion for necessity (Rasoolimanesh et al., 2021; Rasoolimanesh et al., 2023). As shown in Table 7, none of the individual motivational conditions reached this threshold for high sharing intention. Among the positive conditions, tradition motive showed the highest necessity consistency (0.82), followed by altruistic motive (0.80), aesthetic motive (0.79), commercial motive (0.79), hedonistic motive (0.79), affiliation motive (0.73), and recognition motive (0.74). These results indicate that no single motivational condition was individually necessary for high ICHK-sharing intention.

**Table 7:** Necessity analysis for high and low ICHK-sharing intention.

Condition variable	INT		~INT	
	Consistency	Coverage	Consistency	Coverage
AESM	0.79	0.85	0.60	0.49
~AESM	0.53	0.64	0.82	0.75
AFFM	0.73	0.88	0.59	0.53
~AFFM	0.61	0.67	0.87	0.70
ALTM	0.80	0.86	0.62	0.50
~ALTM	0.53	0.65	0.82	0.76
COMM	0.79	0.86	0.63	0.52
~COMM	0.56	0.67	0.83	0.75
HEDM	0.79	0.80	0.64	0.49
~HEDM	0.50	0.65	0.74	0.72
RECM	0.74	0.90	0.55	0.50
~RECM	0.59	0.63	0.89	0.72
TRAM	0.82	0.83	0.64	0.49
~TRAM	0.50	0.65	0.78	0.76

‘∼’ represents the ‘negation’ of a logical operation. AESM, aesthetic motive; AFFM, affiliation motive; ALTM, altruistic motive; COMM, commercial motive; HEDM, hedonistic motive; RECM, recognition motive; TRAM, tradition motive; INT, intention to share ICHK on Douyin.

#### 6.2.2 Sufficient analysis

A truth table was constructed using a frequency threshold of three cases and a raw-consistency threshold of 0.80 (Fiss, 2011; Rasoolimanesh et al., 2021). PRI consistency was also considered using a threshold of 0.8 (Thiem, 2017). Truth-table rows meeting the predefined criteria were retained for logical minimization. The resulting intermediate solution identified nine sufficient configurations associated with high ICHK-sharing intention. The overall solution consistency was 0.96 and the solution coverage was 0.69, indicating that the identified configurations displayed a high degree of subset consistency with the high-intention outcome and jointly covered a substantial proportion of cases belonging to that outcome set.

The nine configurations are summarized in Table 8. Their individual consistencies ranged from 0.96 to 0.97, while raw coverage ranged from 0.35 to 0.50. Unique coverage values were relatively small, ranging from 0.00 to 0.03, reflecting substantial overlap among the sufficient configurations.

**Table 8:** Sufficient configurations associated with high intention to share ICHK on Douyin.

Configuration	Solution								
	1	2	3	4	5	6	7	8	9
AESM		●	●	●	●	●			●
AFFM	●	⊗	●		●		●		●
ALTM	●	●		●		●		●	●
COMM	●		●	●			●	●	●
HEDM		●	●		●	●	●	●	●
RECM				●	●	●	●	●	●
TRAM	●	●	●	●	●	●	●	●	
Consistency	0.96	0.97	0.97	0.97	0.97	0.97	0.97	0.97	0.97
Raw coverage	0.50	0.35	0.46	0.49	0.45	0.44	0.45	0.48	0.41
Unique coverage	0.03	0.03	0.00	0.01	0.01	0.01	0.01	0.00	0.01
Solution consistency	0.96								
Solution coverage	0.69								

“●” indicates the presence of a condition; “⊗” indicates the absence; Blank spaces indicate “do not care.” AESM, aesthetic motive; AFFM, affiliation motive; ALTM, altruistic motive; COMM, commercial motive; HEDM, hedonistic motive; RECM, recognition motive; TRAM, tradition motive; INT, intention to share ICHK on Douyin.

The identified configurations were:

C1: AFFM * ALTM * COMM * TRAM

C2: AESM * ~ AFFM * ALTM * HEDM * TRAM

C3: AESM * AFFM * COMM * HEDM * TRAM

C4: AESM * ALTM * COMM * RECM * TRAM

C5: AESM * AFFM * HEDM * RECM * TRAM

C6: AESM * ALTM * HEDM * RECM * TRAM

C7: AFFM * COMM * HEDM * RECM * TRAM

C8: ALTM * COMM * HEDM * RECM * TRAM

C9: AESM * AFFM * ALTM * COMM * HEDM * RECM.

*Pattern 1: social–instrumental–tradition configurations*

Configurations 1 and 3 share the simultaneous presence of affiliation, commercial, and tradition motives. These configurations suggest that social connection, instrumental return, and cultural or normative commitment can coexist within high sharing intention. In Configuration 3, aesthetic and hedonistic motives are additionally present, indicating a more expressive and enjoyment-oriented variant of the same broader motivational pattern.

*Pattern 2: aesthetic–altruistic–tradition configurations*

Configurations 2 and 4 share the presence of aesthetic, altruistic, and tradition motives, indicating a pattern in which cultural expression, helping others, and normative commitment jointly characterize high sharing intention. Configuration 2 additionally includes hedonistic motive and the absence of affiliation motive, whereas Configuration 4 combines this pattern with commercial and recognition motives. The contrast between the two configurations illustrates equifinality: similar levels of high intention can arise through different accompanying social and instrumental conditions.

*Pattern 3: hedonistic–recognition configurations*

Configurations 5–8 share the joint presence of hedonistic and recognition motives together with tradition motive, while Configuration 9 also contains hedonistic and recognition motives but treats tradition as a “do not care” condition. These solutions indicate that enjoyment and social recognition frequently occur as part of broader motivational combinations associated with high sharing intention, rather than functioning as independently sufficient motives.

Hedonistic motive appeared in seven of the nine sufficient configurations, despite having no significant net association in the PLS-SEM model. Tradition motive was present in eight of the nine sufficient configurations, making it the most recurrent condition in the baseline solution. However, its necessity consistency was only 0.82, below the 0.90 threshold, so it should not be interpreted as a necessary condition. Across the baseline solution, tradition motive appeared in eight configurations, hedonistic motive in seven, aesthetic, altruistic, commercial, and recognition motives in six each, and affiliation motive in five. The absence of affiliation motive appeared in Configuration 2. These frequencies should be interpreted descriptively rather than as evidence of isolated importance because fsQCA evaluates conditions conjuncturally.

Robustness checks were conducted using alternative calibration anchors (90th/50th/10th percentiles) and a stricter raw-consistency threshold of 0.85. The resulting solutions were substantively consistent with the baseline analysis, with only minor differences in individual configurations. The principal motivational patterns, as well as overall solution consistency and coverage, remained stable across specifications.

## 7 Discussion

This study examined youths’ intention to share ICHK on Douyin from two complementary analytical perspectives. The PLS-SEM results indicate that aesthetic, altruistic, commercial, affiliation, tradition, and recognition motives are positively associated with sharing intention, whereas hedonistic motive shows no significant independent net association. The fsQCA results, in contrast, identify multiple motivational configurations associated with high sharing intention. Taken together, the findings indicate that motivational relevance cannot be reduced to whether a single motive has a statistically significant average association with the outcome; some motives also acquire relevance through the combinations in which they occur.

At the variable-centered level, the results suggest that ICHK sharing is associated with multiple forms of motivation rather than a single intrinsic–extrinsic distinction (Maheshwari et al., 2020; Liu and Luo, 2023; Xie et al., 2024; Xu et al., 2020; Zhao and Detlor, 2023). Aesthetic motive reflects the expressive and visual character of communicating cultural knowledge; altruistic motive reflects the perceived value of helping others understand heritage; affiliation and recognition capture different social outcomes of sharing; commercial motive reflects instrumental return; and tradition motive reflects normative and cultural commitment. The simultaneous significance of these motives is consistent with the culturally and socially embedded nature of ICHK sharing, where expressive, prosocial, relational, reputational, instrumental, and normative considerations may coexist within the same behavioral domain.

The nonsignificant PLS-SEM coefficient for hedonistic motive is especially informative. Hedonistic motive showed virtually no independent net association with sharing intention (*β* = 0.001, *p* = 0.484), despite prior research linking enjoyment to online participation and knowledge-sharing activities. This result suggests that enjoyment alone may not distinguish young users with stronger ICHK-sharing intention once the other motivational predictors are considered simultaneously. In the context of cultural heritage knowledge, sharing may involve responsibilities, meanings, and expected social outcomes that extend beyond enjoyment itself. However, this interpretation should not be taken to mean that hedonistic motivation is irrelevant, because the configurational results provide a different picture.

The fsQCA results clarify this distinction. Hedonistic motive appears in seven of the nine sufficient configurations, even though it is nonsignificant in the PLS-SEM model. This is the clearest illustration of the difference between independent net relevance and configurational relevance. Hedonistic motivation may contribute little as an isolated statistical predictor but become relevant when it co-occurs with recognition, tradition, aesthetic, affiliation, altruistic, or commercial motives. The finding therefore cautions against interpreting a nonsignificant net coefficient as evidence of theoretical irrelevance. A second cross-method pattern concerns tradition motive. Its PLS-SEM coefficient is positive but comparatively modest (*β* = 0.134, *p* = 0.003), whereas it appears in eight of the nine baseline fsQCA configurations. At the same time, its necessity consistency for high sharing intention is 0.82, below the 0.90 criterion. Tradition motive should therefore be interpreted as highly recurrent but not necessary. This distinction is theoretically important: a culturally or morally oriented commitment appears frequently within high-intention combinations, but high sharing intention can still occur through at least one configuration in which tradition is not required. Altruistic motive presents the reverse analytical pattern. It has the largest positive PLS-SEM coefficient (*β* = 0.264, *p* < 0.001), yet it appears in only six of the nine baseline configurations and does not satisfy the criterion for necessity. A relatively strong average association therefore does not imply that a motive must be present across all high-intention configurations. Similarly, affiliation motive is positively associated with intention in PLS-SEM (*β* = 0.124, *p* < 0.001), but Configuration 2 explicitly includes the absence of affiliation motive. This indicates that high sharing intention can emerge even under low affiliation when other motives compensate for its absence.

These cross-method differences support an equifinal interpretation of ICHK-sharing motivation. Young users need not arrive at high sharing intention through a uniform motivational profile. Some configurations combine social connection, instrumental return, and cultural commitment; others combine aesthetic expression, altruism, and tradition; still others repeatedly combine enjoyment and recognition with additional cultural, social, or instrumental conditions. The value of the dual-method design therefore lies not in requiring PLS-SEM and fsQCA to produce identical findings, but in showing how average net associations and conjunctural motivational patterns provide different forms of evidence about the same behavioral intention.

### 7.1 Theoretical implications

The study contributes theoretically in three related areas.

First, it contributes to motivation theory by showing that the behavioral salience of broad motivational dispositions is context-dependent. The MVPI provides a general taxonomy of valued outcomes, but the present findings suggest that their relevance to a specific behavior depends on the knowledge domain and communication context in which they are enacted. This is evident both in the qualitative pretest, which identified only seven contextually salient MVPI domains, and in the quantitative findings, where those motives displayed different forms of net and configurational relevance. The contribution is therefore not the creation of new motivational dimensions, but the demonstration that broad motivational dispositions require contextual interpretation when applied to culturally embedded digital practices.

Second, the study contributes to digital media psychology by conceptualizing platform characteristics as conditions for motivational enactment rather than as sources that create motivation. Douyin’s audiovisual format, visible audience feedback, interaction mechanisms, and creator-oriented economic environment provide different opportunities through which aesthetic, recognition, affiliation, commercial, and hedonistic motives may be expressed. Importantly, these platform characteristics were not modeled as mediators or moderators in the present study. The findings therefore do not establish that algorithmic distribution changes users’ underlying motives. Instead, they suggest that a platform environment can recontextualize the conditions under which existing motives become behaviorally salient.

Third, the study contributes to cultural heritage communication by showing that digital ICHK sharing involves motivational hybridity. Heritage communication is not explained solely by preservation-oriented or culturally normative motivations. Tradition and altruism coexist with aesthetic expression, social affiliation, recognition, enjoyment, and commercial considerations. The fsQCA results make this particularly visible because high sharing intention emerges through multiple combinations that mix culturally oriented and platform-oriented motives. This suggests that participation in digital heritage communication should not be conceptualized as a simple opposition between cultural commitment and contemporary platform incentives; the two can coexist within the same motivational configuration.

Methodologically, the integration of PLS-SEM and fsQCA further contributes by distinguishing independent net relevance from configurational relevance. A motive can have a strong net association without being necessary across configurations, as illustrated by altruistic motive, while another motive can have little independent net association but recur across multiple sufficient configurations, as illustrated by hedonistic motive. This distinction provides a more precise account of motivational heterogeneity than either analytical perspective would provide in isolation.

### 7.2 Practical implications

The findings suggest that interventions designed to support ICHK sharing should not rely on a single universal motivational appeal. The PLS-SEM results indicate that several motives are independently associated with sharing intention, while the fsQCA results show that high intention can emerge through different motivational combinations. Cultural authorities, ICH institutions, and platform practitioners should therefore consider differentiated intervention designs that align with distinct motivational profiles rather than assuming that all young users respond to the same incentive.

One intervention pathway can combine aesthetic, altruistic, and tradition-oriented appeals. Cultural institutions can provide short-video storytelling templates, visual-production guidance, concise heritage-information resources, and access to expert or inheritor consultation. Such support would allow young creators to present culturally accurate information while also making heritage content visually intelligible and useful to others. Campaigns organized around cultural explanation, intergenerational transmission, or “help others understand this tradition” can align aesthetic expression with prosocial and cultural-responsibility motives.

A second pathway can combine tradition, affiliation, and commercial motives. Institutions can establish creator communities, creator–inheritor co-production programmes, peer-review networks, workshops, micro-grants, or legitimate cultural-product and tourism partnerships. In this configuration, commercial incentives should be tied to culturally responsible production rather than traffic volume alone. Material support can therefore function alongside community participation and cultural commitment without requiring commercial motivation to displace heritage values.

A third pathway concerns the recurrent combination of hedonistic and recognition motives. Because hedonistic motive was nonsignificant as an independent net predictor but appeared repeatedly in the fsQCA configurations, entertainment-oriented design should not be treated as a sufficient strategy by itself. Instead, playful storytelling, creative challenges, audiovisual experimentation, or participatory formats can be combined with forms of culturally meaningful recognition. These might include expert-reviewed selections, thematic featuring, creator–inheritor co-credit, public exhibitions, or heritage-contributor recognition. Such mechanisms provide acknowledgement without reducing recognition solely to likes, follower counts, or traffic metrics.

The recurrence of tradition motive across most baseline configurations also suggests that culturally meaningful framing can function as a cross-cutting element in many interventions, although it should not be treated as universally necessary. Different motivational combinations remain viable, and a single intervention model is therefore unlikely to fit all young users.

For platform practitioners, the findings support the development of differentiated creator-support mechanisms, heritage-themed spaces, co-creation opportunities, contextual information prompts, and recognition systems for culturally responsible contributions. Because algorithmic recommendation mechanisms were not directly measured in this study, the findings do not justify specific claims about how ranking or recommendation systems should be altered. Future platform experiments could test whether changes in visibility, feedback, recognition, or monetization features modify actual heritage-sharing behavior.

### 7.3 Limitations and further research

Several limitations define the boundaries of the present findings. First, the study focuses on behavioral intention rather than observed sharing behavior. Intention provides an important proximal indicator of participation, but users who intend to share cultural knowledge may not ultimately do so. Future research could combine survey measures with behavioral indicators such as actual posting, forwarding, commenting, content production, or longitudinal creator activity.

Second, the motivational framework was contextually delimited on the basis of the qualitative pretest. Scientific, power, and security motives were not spontaneously salient among the 24 pretest participants and were therefore not included in the formal model. This decision improved alignment between the empirical model and the focal context but does not imply that these motives are irrelevant to ICHK sharing in other settings. Scientific motivation may become more salient in documentation or research-oriented heritage contexts, security concerns may matter where cultural or intellectual property is contested, and power-related motives may emerge where authority over cultural representation is more prominent. Cross-platform and cross-context studies are needed to examine these boundary conditions.

Third, the non-probability snowball sampling design limits population-level generalizability. Recruitment through university associations, volunteer networks, and cultural-interest groups may have overrepresented respondents who were more socially connected, digitally active, or culturally engaged than the broader youth population. Equal quotas ensured balanced representation of the four study regions within the analytical sample but did not correct referral-chain homophily or reproduce the underlying population distribution. Future studies should consider probability-based, stratified, or larger multi-site sampling designs.

Fourth, the study was limited to young people aged 18–24 and to Douyin users in four regions of Sichuan. The motivational patterns identified here may differ among older users, less digitally active populations, other cultural regions, or users of platforms with different affordance structures. Comparative studies across Douyin, WeChat, Bilibili, Xiaohongshu, TikTok, Instagram, or other platforms could provide stronger evidence about which motivational patterns are platform-specific and which are more generalizable.

Finally, the present study integrates variable-centered and configurational analyses, but the model does not directly measure platform affordances, cultural identity, perceived authenticity, or other psychological mechanisms that might explain why specific motivational combinations become salient. Future research could extend the framework by incorporating these constructs as theoretically justified antecedents, moderators, or mediating mechanisms, provided that such extensions are tested with designs capable of supporting the corresponding interpretations.

## 8 Conclusion

This study examined the motivational structure associated with young people’s intention to share ICHK on Douyin by integrating PLS-SEM and fsQCA. The PLS-SEM results indicate that aesthetic, altruistic, commercial, affiliation, tradition, and recognition motives are positively associated with sharing intention, whereas hedonistic motive shows no significant independent net association. The fsQCA analysis provides a complementary perspective by identifying multiple equifinal configurations associated with high sharing intention. No single motivational condition was individually necessary for the outcome. Importantly, hedonistic motive appeared in seven of the nine baseline sufficient configurations despite its nonsignificant PLS-SEM coefficient, while tradition motive appeared in eight configurations without meeting the criterion for necessity. These findings illustrate that independent net relevance and configurational relevance are analytically distinct: a motive may contribute little as an isolated predictor yet remain salient when combined with other motivational conditions. Taken together, the findings support a context-sensitive and conjunctural understanding of motivation in digital heritage communication. Young people may arrive at similarly high ICHK-sharing intention through different combinations of cultural, social, expressive, reputational, hedonic, and instrumental motives. The study therefore extends research on social-media knowledge sharing by showing that motivational relevance depends not only on individual predictors but also on the combinations in which motives occur. These findings should be interpreted within the boundaries of the research design. The data are cross-sectional and self-reported, the outcome is behavioral intention rather than observed sharing behavior, and the snowball sampling procedure limits population-level generalizability. Accordingly, the reported relationships should be interpreted as statistical and set-theoretic associations rather than established causal effects. Future longitudinal, experimental, and behavioral studies could examine how these motivational patterns develop over time and whether specific platform conditions alter actual ICHK-sharing behavior.

## Data Availability

The original contributions presented in the study are included in the article/Supplementary material, further inquiries can be directed to the corresponding author.
