# Supplementary material for: Motivational predictors and configurations associated with youths’ intention to share intangible cultural heritage knowledge on Douyin: a PLS-SEM and fsQCA study
Source: Front Psychol. 2026 Oct 20;17:1935285. doi: 10.3389/fpsyg.2026.1935285 (PMC13735965; doi:10.3389/fpsyg.2026.1935285)
Supplement: Supplementary file 1 [file Supplementary_file_1.DOCX]

**Supplementary Information**

**TABLE A1** Definition of the ten motivations of MVPI.

| **Variables** | **Descriptions** |
| --- | --- |
| Aesthetic motive | A necessity for self-expression, coupled with an inclination towards entities' aesthetics, tactile qualities, and functional attributes. |
| Altruistic motive | An inclination towards altruism, accompanied by a compassionate regard for the well-being of individuals less advantaged in societal circumstances. |
| Commercial motive | An engagement with economic pursuits, encompassing the pursuit of financial gain, profit realisation, and identifying novel avenues for business expansion. |
| Hedonistic motive | The quest for enjoyment, thrill, gratification, and recreational engagement. |
| Power motive | An aspiration for achievement, proactive endeavor, and effecting meaningful change. |
| Recognition motive | A yearning for recognition, visibility, prominence, and renown. |
| Scientific motive | A focus on scientific inquiry, proficiency with technological advancements, and a penchant for data analysis. |
| Security motive | A requirement for predictability and organisational framework, coupled with endeavors to mitigate risk and uncertainty. |
| Affiliation motive | An emphasis on the necessity for and appreciation of diverse and recurrent social interactions. |
| Tradition motive | A commitment to and adherence to principles encompassing familial bonds, religious affiliation, frugality, and industriousness. |

**TABLE A2. Summary of related studies used MVPI.**

| **Authors** | **Study objective** | **MVPI Adoption** | **Study theme** |
| --- | --- | --- | --- |
| Furnham (2015) | To see which bright and dark side traits were related to achievement motivation and how much variance they might account for. | ⍻ | PCV |
| Furnham and Treglown (2021) | To document differences in large groups of adults | ✓ | PCV |
| Romanová et al. (2016) | To verify the validity of MVPI | ✓ | PCV |
| Furnham and Treglown (2022) | To detect the differences between those who choose scientific jobs over business/practitioner jobs and consulting assignments. | ✓ | IDP |
| Furnham et al. (2015) | To examine the correlation of individual differences in the Hogan Personality Inventory effectiveness scale, which measures the extent to which respondents report in a careless, erratic, or random manner. | ✓ | IDP |
| Jordan et al. (2018) | To understand the personality traits of emergency medicine residents and assess consistency and differences between residency programs. | ✓ | IDP |
| Bălan et al. (2019) | To offer an exploratory look at generational differences among Romanian leaders. | ✓ | OBL |
| Furnham et al. (2016) | To test for cross-validation, dark-side traits (derailers) and work values (motives) were examined to determine how they relate to occupational potential in sales and managerial jobs. | ✓ | OBL |
| Noaghea et al. (2017) | To draw personality profiles of Romanian leaders and verify whether there are differences between industries | ✓ | OBL |

OBL: organisational behavior and leadership, IDP: individual differences and personality, PCV: psychometric characteristics and validation. "✓" refers to all the motivation factors adopted from MVPI: aesthetic, altruistic, commercial, hedonistic, power, recognition, scientific, security, affiliation, and tradition motives. "⍻" refers to part of the motivation factors adopted from MVPI.

**TABLE A3** The adapted items.

| **Construct and scale items** | |
| --- | --- |
| **Aesthetic Motive** | |
| AESM1 | Sharing intangible cultural heritage knowledge (ICHK) on Douyin can describe me. |
| AESM2 | Sharing ICHK on Douyin can affirm my value. |
| AESM3 | Sharing ICHK on Douyin can make me have strong feelings. |
| **Affiliation Motive** | |
| AFFM1 | I think being close to the other Douyin members by sharing ICHK on Douyin, listening to them, and connecting with them on an interactive level is one of my favourite and satisfying pastimes. |
| AFFM2 | One of the most exciting things I can think of is connecting with and getting to know other Douyin members by sharing ICHK on Douyin. |
| AFFM3 | When I could get close to other Douyin members by sharing my ICHK on Douyin, I felt that I had accomplished something valuable. |
| AFFM4 | If I can make new friends with my favourite Douyin members by sharing my ICHK on Douyin, I will feel delighted. |
| **Altruistic Motive** | |
| ALTM1 | I like helping others on Douyin. |
| ALTM2 | Sharing my ICHK on Douyin can help others with similar interests. |
| ALTM3 | I enjoy helping others by sharing my ICHK on Douyin. |
| **Commercial Motive** | |
| COMM1 | I will receive monetary rewards in return for sharing my ICHK on Douyin. |
| COMM2 | I will receive additional incentives related to ICH in return for sharing my ICHK on Douyin. |
| COMM3 | How hard I work on ICHK sharing on Douyin is directly linked to how much I will be rewarded. |
| **Hedonistic Motive** | |
| HEDM1 | While participating in sharing ICHK on Douyin, I will experience pleasure. |
| HEDM2 | The process of participating in sharing ICHK on Douyin will be exciting. |
| HEDM3 | I have fun using Douyin to share my ICHK. |
| **Recognition Motive** | |
| RECM1 | Sharing my ICHK on Douyin improves my image on Douyin. |
| RECM2 | Users in Douyin who share their ICHK have more prestige than those who do not. |
| RECM3 | Sharing my ICHK on Douyin will improve others’ recognition of me. |
| RECM4 | When I share my ICHK on Douyin, the other users will respect me. |
| RECM5 | When I share my ICHK on Douyin, maybe people who are important to me will praise me. |
| **Tradition Motive** | |
| TRAM1 | My conscience calls me to contribute my ICHK on Douyin. |
| TRAM2 | Whether or not to share ICHK on Douyin aligns with my moral conviction. |
| TRAM3 | I feel morally obliged to share my ICHK on Douyin. |
| **Intention to Share ICH** **Knowledge on Douyin** | |
| INT1 | I intend to share my ICHK through Douyin. |
| INT2 | I will try to share my ICHK through Douyin. |
| INT3 | I plan to share my ICHK through Douyin. |
